# The Good, the Bad, and the Deadly: Adenosinergic Mechanisms Underlying Sudden Unexpected Death in Epilepsy

**DOI:** 10.3389/fnins.2021.708304

**Published:** 2021-07-12

**Authors:** Benton Purnell, Madhuvika Murugan, Raja Jani, Detlev Boison

**Affiliations:** ^1^Department of Neurosurgery, Robert Wood Johnson Medical School, Rutgers University, Piscataway, NJ, United States; ^2^Rutgers Neurosurgery H.O.P.E. Center, Department of Neurosurgery, Rutgers University, New Brunswick, NJ, United States; ^3^Brain Health Institute, Rutgers University, Piscataway, NJ, United States

**Keywords:** adenosine, epilepsy, SUDEP, status epilepticus, seizure-induced respiratory arrest, adenosine kinase, adenosine receptors, epileptogenesis

## Abstract

Adenosine is an inhibitory modulator of neuronal excitability. Neuronal activity results in increased adenosine release, thereby constraining excessive excitation. The exceptionally high neuronal activity of a seizure results in a surge in extracellular adenosine to concentrations many-fold higher than would be observed under normal conditions. In this review, we discuss the multifarious effects of adenosine signaling in the context of epilepsy, with emphasis on sudden unexpected death in epilepsy (SUDEP). We describe and categorize the beneficial, detrimental, and potentially deadly aspects of adenosine signaling. The good or beneficial characteristics of adenosine signaling in the context of seizures include: (1) its direct effect on seizure termination and the prevention of status epilepticus; (2) the vasodilatory effect of adenosine, potentially counteracting postictal vasoconstriction; (3) its neuroprotective effects under hypoxic conditions; and (4) its disease modifying antiepileptogenic effect. The bad or detrimental effects of adenosine signaling include: (1) its capacity to suppress breathing and contribute to peri-ictal respiratory dysfunction; (2) its contribution to postictal generalized EEG suppression (PGES); (3) the prolonged increase in extracellular adenosine following spreading depolarization waves may contribute to postictal neuronal dysfunction; (4) the excitatory effects of A_2A_ receptor activation is thought to exacerbate seizures in some instances; and (5) its potential contributions to sleep alterations in epilepsy. Finally, the adverse effects of adenosine signaling may potentiate a deadly outcome in the form of SUDEP by suppressing breathing and arousal in the postictal period. Evidence from animal models suggests that excessive postictal adenosine signaling contributes to the pathophysiology of SUDEP. The goal of this review is to discuss the beneficial, harmful, and potentially deadly roles that adenosine plays in the context of epilepsy and to identify crucial gaps in knowledge where further investigation is necessary. By better understanding adenosine dynamics, we may gain insights into the treatment of epilepsy and the prevention of SUDEP.

## Introduction

The purine ribonucleoside adenosine is found ubiquitously in living tissues. In the central nervous system, adenosine is an essential inhibitory modulator of neuronal excitability (Dunwiddie, 1980; Dunwiddie and Masino, 2001; Boison, 2008). Neuronal activity increases adenosine signaling thereby providing negative feedback on excessive excitation (Mitchell et al., 1993; Brager and Thompson, 2003; Pajski and Venton, 2010). The exceptionally high neuronal activity of a seizure results in a surge in extracellular adenosine to concentrations many fold higher than would be observed under normal conditions (During and Spencer, 1992; Berman et al., 2000; Van Gompel et al., 2014). The neuronal inhibition provided by activity-dependent adenosine surging is critical to the prevention and termination of seizures (Dragunow et al., 1985; Murray et al., 1985; Kochanek et al., 2006). Deficits in adenosine signaling can facilitate status epilepticus, a life-threatening event defined by inordinately protracted seizure activity (Young and Dragunow, 1994; Kochanek et al., 2006). In this sense, seizure-induced adenosine surging is highly beneficial. Seizures can also cause periods of profound cerebral hypoxia through postictal vasoconstriction, increased oxygen demand, and respiratory dysfunction (Posner et al., 1969; Farrell et al., 2017; Lacuey et al., 2018). Cerebral hypoxia likely contributes to the pathophysiology of a number of adverse seizure outcomes such as neurodegeneration, memory loss, postictal generalized EEG suppression (PGES), and the postictal state (Seyal et al., 2012; Farrell et al., 2016; Leal-Campanario et al., 2017; Rheims et al., 2019). Adenosine acts as a cerebral vasodilator and may alleviate the deleterious effects of seizure-induced vasoconstriction (Morii et al., 1986; Arrigoni et al., 2005). Furthermore, adenosine signaling is neuroprotective under hypoxic conditions (Bjorklund et al., 2008; Phillips et al., 2019). As a result, seizure-induced adenosine surging and the increased tissue tone of adenosine are “good” for patient health.

On the other hand, large surges in extracellular adenosine can have detrimental effects. Excessive increases in extracellular adenosine suppress neuronal activity and may contribute to PGES and the postictal state (Rosen and Berman, 1985; During and Spencer, 1992). Though acute seizures cause an increase in extracellular adenosine, chronic epilepsy is associated with a reduction of baseline adenosine levels, which could be a precipitating factor in epileptogenesis (Gouder et al., 2004; Li et al., 2008) and associated co-morbid conditions including cognitive, psychiatric, and sleep disorders (Yee et al., 2007; Boison et al., 2012; Shen et al., 2012; Boison, 2016; Warren et al., 2018). Spreading depolarization waves, which can occur during seizures, result in a prolonged increase in extracellular adenosine (Lindquist and Shuttleworth, 2014; Loonen et al., 2019). This increase in adenosine contributes to the neuronal dysfunction that persists in the wake of a spreading depolarization wave (Lindquist and Shuttleworth, 2017). Additionally, under certain circumstances, A_2A_ receptor activation may have proconvulsant effects (Zeraati et al., 2006; Fukuda et al., 2011); however, anticonvulsant effects of A_2A_ receptor activation have also been described (De Sarro et al., 1999; Huber et al., 2002). Of concern, adenosine suppresses breathing and attenuates the hypercapnic ventilatory response through inhibition of brainstem respiratory sites (Gettys et al., 2013; Falquetto et al., 2018). Seizure-induced increases in brainstem adenosine levels may make seizures more dangerous by preventing an adequate respiratory response to postictal blood gas derangement. For these reasons, seizure-induced adenosine surging is “bad” for patient health.

The adverse effects of seizure-induced adenosine surging may play a critical role in seizure-induced death. The leading cause of epilepsy-related death in patients with refractory epilepsy is sudden unexpected death in epilepsy (SUDEP; Hesdorffer et al., 2011). More years of potential life are lost due to SUDEP than any other neurological condition with the exception of stroke (Thurman et al., 2014). Currently, there are no reliable means of preventing SUDEP or identifying those who are at the highest risk (Massey et al., 2014; Devinsky et al., 2016; Dlouhy et al., 2016). Convergent lines of evidence from epilepsy patients and animal models suggests that SUDEP is the result of some combination of respiratory, cardiac, and electrocerebral dysfunction in the postictal period (Jehi and Najm, 2008; Massey et al., 2014; Aiba and Noebels, 2015; Dlouhy et al., 2016). The precise pathophysiology of SUDEP is the subject of a vibrant ongoing debate (Auerbach et al., 2013; Aiba and Noebels, 2015; Budde et al., 2018; Vega, 2018; Vilella et al., 2019). Currently, the most reliable information on the terminal cascade which precedes SUDEP comes from a case series of SUDEP events occurring in epilepsy monitoring units in which video, electrocardiogram (EKG), and electroencephalogram (EEG) were simultaneously recorded (Ryvlin et al., 2013). In all cases in which breathing and cardiac function could be assessed, terminal apnea preceded terminal asystole indicating that respiratory failure was the primary cause of death (Ryvlin et al., 2013). In the forebrain, seizure-induced adenosine surging has the beneficial effect of stopping seizures; however, excessive adenosine signaling may potentiate SUDEP through respiratory suppression, PGES exacerbation, and attenuation of the hypercapnic ventilatory response (Shen et al., 2010; Ashraf et al., 2020). Experimental evidence from animal models of seizure-induced death suggests that excessive adenosinergic signaling contributes to SUDEP pathophysiology (Shen et al., 2010; Fukuda et al., 2011; Faingold et al., 2016; Kommajosyula et al., 2016). Hence, seizure-induced adenosine surging must be tightly controlled to prevent the potentially disastrous outcomes of status epilepticus and SUDEP. In this review, we summarize the evidence pertinent to the adenosine hypothesis of SUDEP and describe the multifarious effects of adenosine signaling in the context of epilepsy: the good, the bad, and the deadly.

## The Good

### Adenosine and Seizure Cessation

**FIGURE 1:**
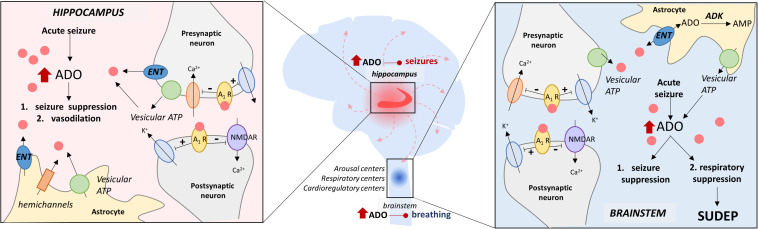
The adenosine hypothesis of SUDEP. This schematic illustrates the hypothesized similarities and differences in periictal adenosine surging and its effects in the hippocampus and the brainstem. In the hippocampus, the ADO surge is important for seizure termination and vasodilation, whereas, in the brainstem, the ADO surge is double-edged and can suppress respiration/breathing in addition to seizure suppression. The key elements of ADO signaling depicted here include (i) vesicular ATP, (ii) equilibrative nucleoside transporters (ENT), (iii) adenosine kinase (ADK), (iv) adenosine receptor (A_1_R), and (v) N-methyl D-aspartate receptors (NMDAR).

In the central nervous system, adenosine is released in neural tissue in response to endogenously generated activity (Mitchell et al., 1993; Nguyen and Venton, 2015). Exogenously evoked neuronal activity also triggers an increase in extracellular adenosine (Sulakhe and Phillis, 1975; Lloyd et al., 1993; Pajski and Venton, 2010; Tawfik et al., 2010). The magnitude of the activity-dependent adenosine release increases with the intensity of stimulation (Mitchell et al., 1993; Sciotti et al., 1993; Pajski and Venton, 2010). The high amplitude and high frequency firing of epileptiform discharges results in more neuronal activity than would occur under normal conditions (Merricks et al., 2015). Therefore, it is unsurprising that seizures result in a surge in extracellular adenosine to concentrations much higher than those seen under normal conditions (Figure 1; During and Spencer, 1992; Berman et al., 2000; Aden et al., 2004). Adenosine is an inhibitory modulator of presynaptic neurotransmission and activity dependent adenosine release is critical to keeping neural excitability in check (Dunwiddie and Masino, 2001).

Interestingly, the source of activity-dependent adenosine release remains elusive. There are a number of potential sources of extracellular adenosine (Latini and Pedata, 2001; Wall and Dale, 2008). The mechanism responsible for activity dependent adenosine release varies depending on the brain region (Pajski and Venton, 2013) and the parameters of neural activity (Cunha et al., 1996). Furthermore, activity-dependent adenosine release can occur through several mechanisms simultaneously. A study with transgenic mice with an inducible astrocyte-selective mutation of soluble N-ethylmaleimide-sensitive factor attachment protein receptor (dnSNARE-mice) suggests that astrocytic vesicular release of adenosine triphosphate (ATP) is the major source of synaptic adenosine (Pascual et al., 2005). On the other hand, hippocampal neuronal activity results in increased extracellular adenosine *via* a combination of astrocytic ATP release, and neuronal adenosine release through equilibrative nucleoside transporters (Wall and Dale, 2013). Contrary to this, another study showed that blocking the conversion of ATP to adenosine did not alter the inhibition of neuronal activity associated with high frequency stimulation, suggesting that neuronal adenosine release, and not astrocytic ATP release mediated feedback inhibition of excitatory activity (Lovatt et al., 2012).

Whatever the source of extracellular adenosine, the breakdown is controlled by intracellular astrocytic adenosine kinase (ADK); thus, the tone of ambient adenosine is maintained by an astrocyte-based adenosine-cycle (Fredholm et al., 2005). It is noteworthy, that during chronic epileptic conditions ADK expression is upregulated and consequently the tissue tone of adenosine is drastically reduced (Boison, 2012). Hence, the seizure-induced surge of adenosine combined with a low basal level of adenosine creates a complex reperfusion scenario that needs to be investigated particularly in the context of SUDEP.

Adenosine receptors are G-protein-coupled and exert their effects on neuronal excitability through several transduction pathways. A_1_ receptors, which are coupled to G_*i/o*_ proteins (Fredholm et al., 2011), hyperpolarize neurons by activating potassium channels (Figure 1; Trussell and Jackson, 1985) and inhibiting voltage dependent calcium channels (Figure 1; MacDonald et al., 1986). On the other hand, A_2A_ receptors, which are coupled to G_*s/olf*_ proteins (Fredholm et al., 2011), are linked to adenylyl cyclase activation and are thought to have an excitatory effect on neurons upon activation (Corvol et al., 2001).

**FIGURE 2:**
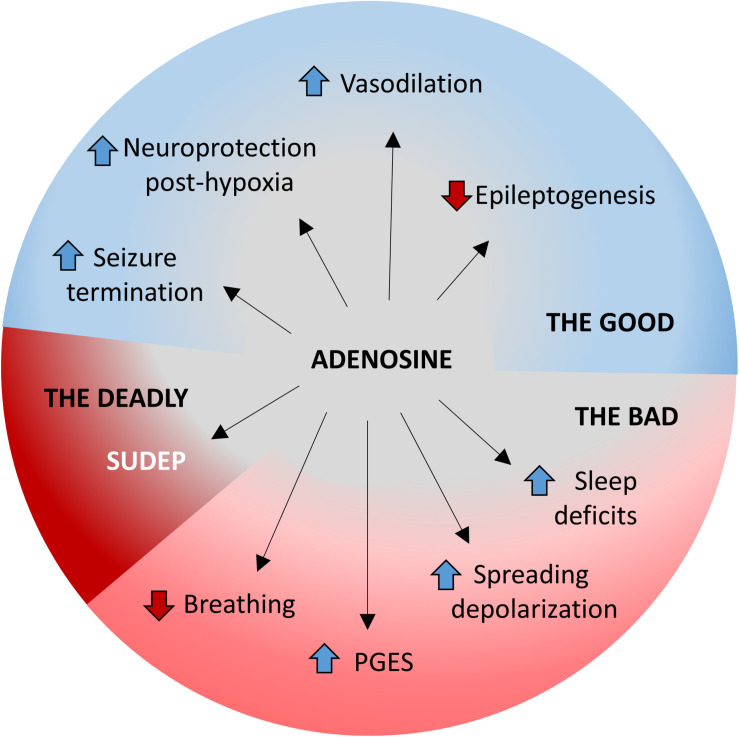
The good, the bad, and the deadly effects of adenosine during seizures. The multifaceted roles of adenosine homeostasis during seizures is highlighted. The beneficial/good aspects include (i) seizure termination, (ii) neuroprotection against hypoxia, (iii) increased vasodilation, and (iv) prevention of epileptogenesis. The detrimental/bad aspects include (i) suppression of breathing, (ii) PGES, (iii) worsened electrographic dysfunction following spreading depolarization, and (iv) development of sleep deficits. Excessive adenosine release or insufficient adenosine clearance may result in a deadly outcome in the form of SUDEP.

Activity-dependent adenosine release alters seizure dynamics largely *via* interactions with the A_1_R. Reducing the influence of activity dependent adenosine release *via* A_1_R antagonists prolongs seizures in animal models (Dragunow and Goddard, 1984). Likewise, the genetic deletion of the A_1_R increases vulnerability to status epilepticus and traumatic brain injury (Fedele et al., 2006; Kochanek et al., 2006). Conversely, upregulating adenosinergic tone by inhibiting adenosine reuptake or degradation is protective against seizure activity (Dragunow and Goddard, 1984; Gouder et al., 2004). Stem cell derived implants with deficient adenosine metabolism suppressed seizures in rats (Guttinger et al., 2005). Those findings from rodent models are relevant for the human brain as upregulation of adenosine signaling in excised human epileptic tissue attenuated spontaneous and evoked epileptiform activity (Kostopoulos et al., 1989). Conversely, the adenosine receptor antagonist caffeine is used clinically for the purpose of prolonging seizure duration following electroconvulsive therapy, ostensibly improving treatment outcomes (Bozymski et al., 2018). In summary, the inhibitory influence of adenosine is critical to seizure termination (Figure 2 and Table 1).

**TABLE 1:** The beneficial effects of adenosine in the context of seizures and epilepsy.

**The Good:** Adenosine increases seizure threshold, is critical for seizure termination, and may alleviate some of the adverse effects of seizures.	

Seizure cessation	The inhibitory influence of adenosine makes seizures less likely and is critical for preventing status epilepticus when seizures do occur.
Neuroprotection during hypoxia	Convergent lines of evidence indicate that adenosine is neuroprotective under hypoxic conditions such as those observed during seizures.
Vasodilation	The vasodilating effect of seizure-induced adenosine surging may attenuate the postictal dysfunction elicited by cerebral vasoconstriction.

### Adenosine Is Neuroprotective Under Hypoxic Conditions

The metabolic demand associated with seizures increases O_2_ consumption and CO_2_ production (Posner et al., 1969). Insufficient blood gas exchange due to cerebral vasoconstriction, increased metabolism, and seizure-induced respiratory dysfunction contribute to cerebral hypoxia (Posner et al., 1969; Farrell et al., 2017; Lacuey et al., 2018). Reduction in cerebral O_2_ to less than half of baseline levels can occur within seconds of seizure termination and can last more than an hour (Farrell et al., 2016). Cerebral hypoxia is associated with PGES and the postictal state (Farrell et al., 2016; Kuo et al., 2016; Rheims et al., 2019). Seizure-induced cerebral hypoxia can also contribute to memory disruption (Farrell et al., 2020).

Convergent lines of evidence indicate that adenosine is neuroprotective under hypoxic conditions (Figure 2 and Table 1). Adenosine and A_1_ receptor agonists alleviate hypoxic/ischemic damage in cultured cells (Goldberg et al., 1988; Daval and Nicolas, 1994), in isolated slice preparations (Dux et al., 1992; Mori et al., 1992), and *in vivo* (Evans et al., 1987; von Lubitz et al., 1988; Von Lubitz et al., 1994). Likewise, non-selective and A_1_ receptor specific adenosine antagonists exacerbate hypoxic/ischemic damage in cultured cells (Daval and Nicolas, 1994; Lynch et al., 1998), in isolated slice preparations (Dux et al., 1992), and *in vivo* (Von Lubitz et al., 1994). The neuroprotective effect of A_1_R mediated adenosine signaling has also been demonstrated in the retina (Larsen and Osborne, 1996). Furthermore, increasing adenosine signaling by reducing its metabolic clearance through inhibition of adenosine deaminase (Phillis and O’Regan, 1989; Lin and Phillis, 1992) or ADK (Miller et al., 1996; Jiang et al., 1997) protects against hypoxic/ischemic damage. Lastly, increasing adenosine signaling by inhibiting its reuptake is neuroprotective under hypoxic/ischemic conditions (DeLeo et al., 1988; Matsumoto et al., 1996).

Though A_1_ receptor activation is neuroprotective under hypoxic conditions, the opposite appears to be true of the A_2A_ receptor. A_1_ receptor knockout mice are more vulnerable to hypoxic/ischemic damage (Johansson et al., 2001), whereas the converse is true in A_2A_ receptor knockout mice (Chen et al., 1999). A_2A_ receptor antagonists alleviate hypoxic/ischemic damage *in vivo* (Gao and Phillis, 1994; Phillis, 1995; Von Lubitz et al., 1995) and the A_2A_ receptor antagonist KW-6002 is now FDA approved as an adjunct treatment for Parkinson’s disease (Berger et al., 2020; Chen and Cunha, 2020).

### Adenosine-Induced Vasodilation

Cerebral vasoconstriction has been documented in the postictal period in epilepsy patients and in animal models (Newton et al., 1992; Steinhoff et al., 1996; Farrell et al., 2016, 2017). There are several ways in which postictal vasoconstriction might contribute to adverse seizure sequelae. Firstly, vasoconstriction contributes to postictal cerebral hypoxia (Farrell et al., 2016, 2017). As mentioned in the preceding section, postictal cerebral hypoxia has been associated with PGES, the postictal state, and seizure-induced memory impairments (Farrell et al., 2016; Kuo et al., 2016). Secondly, the hypoperfusion caused by repeated seizures may contribute to progressive neurodegeneration (Leal-Campanario et al., 2017). Fortunately, adenosine is known to act as a vasodilator in the central nervous system (Morii et al., 1986; Arrigoni et al., 2005) and is released in large quantities during seizures (During and Spencer, 1992; Berman et al., 2000; Van Gompel et al., 2014). The vasodilating effect of seizure-induced adenosine surging may attenuate the postictal dysfunction elicited by cerebral vasoconstriction (Figure 2 and Table 1). This hypothesis is supported by the finding that adenosine antagonism worsens the cerebral hypoxia caused by postictal hypoperfusion (Phillips et al., 2019). Taken together, the seizure-induced adenosine surge is beneficial in its effects on seizure termination, prevention of epileptogenesis, vasodilation, and neuroprotection under hypoxic conditions (Figure 2 and Table 1).

## The Bad

The inhibitory influence of activity dependent adenosine surging is essential to the regulation of neuronal activity and to the prevention and cessation of seizures (Dragunow et al., 1985; During and Spencer, 1992; Young and Dragunow, 1994; Kochanek et al., 2006); however, excessive adenosine signaling can also have detrimental effects including suppression of breathing, PGES, neuronal dysfunction following spreading depolarization, and may even play a contributing role in the development of comorbid conditions (Figure 2 and Table 2).

**TABLE 2:** The potentially harmful effects of adenosine in the context of seizures and epilepsy.

**The Bad:** Adenosine surging due to seizures or to secondary depolarization events may adversely affect breathing and EEG activity acutely and sleep chronically.	

Respiratory suppression	Adenosine suppresses breathing and seizure-induced adenosine surging has been implicated in periictal respiratory dysfunction.
The postictal state and PGES	Excessive increases in extracellular adenosine suppress neuronal activity and may contribute to PGES and the postictal state.
Spreading depolarization	The increase in extracellular adenosine caused by periictal spreading depolarization may contribute to postictal electrocerebral dysfunction.
Proconvulsant effects	Though adenosine is generally inhibitory, there is mixed evidence that under certain circumstances A_2A_ receptor activation can have proconvulsant effects.
Sleep deficits	Adenosinergic dysfunction in chronic epilepsy may contribute to comorbid sleep disorders.

### Adenosine and Breathing

Adenosine suppresses breathing by a reduction of both respiratory rate and volume (Figure 2 and Table 2; Eldridge et al., 1984, 1985; Lagercrantz et al., 1984). Interestingly, in mechanically ventilated cats, intracerebroventricular administration of an adenosine analog suppressed respiratory drive while causing the medulla to become acidified (Eldridge et al., 1984), which would normally be expected to increase respiratory drive (Hodges et al., 2004). Because the animals in this study were mechanically ventilated, the medullary acidosis appears to be metabolic in origin and is not explicable by changes in breathing (Eldridge et al., 1984). In adults, adenosine suppresses breathing *via* its action on the A_1_ receptor (Herlenius et al., 1997; Gettys et al., 2013), but A_2A_-mediated suppression of breathing has been documented in neonates (Koos et al., 2001; Mayer et al., 2006). Increases in adenosine signaling in the nucleus tractus solitarius, the pontine reticular formation, and the pre-Bötzinger complex have all been demonstrated to suppress breathing (Douglas et al., 1982; Yan et al., 1995; Gettys et al., 2013).

Respiratory disruption can result in potentially dangerous derangement of blood gasses. The hypercapnic ventilatory response is a life-saving reflex that increases breathing in response to rising CO_2_ levels (Douglas et al., 1982; Ainslie and Duffin, 2009). In addition to its effect on baseline breathing, adenosine attenuates the hypercapnic ventilatory response (Falquetto et al., 2018). Conversely, adenosine receptor antagonists improve the hypercapnic ventilatory response (Pianosi et al., 1994). Serotonergic neurons in the raphe nuclei are chemosensitive and contribute to the hypercapnic ventilatory response (Hodges et al., 2008; Teran et al., 2014). Serotonin release is inhibited by adenosine agonists and enhanced by adenosine antagonists (Feuerstein et al., 1988; Okada et al., 2001; Arnold et al., 2019). Similarly, the retrotrapezoid nucleus is chemosensitive and may contribute to the hypercapnic ventilatory response (Guyenet et al., 2016). Chemosensitive neurons in the retrotrapezoid nucleus are inhibited by A_1_ receptor activation (James et al., 2018). Increased inhibition of brain areas relevant to respiratory chemoreception, such as the raphe and retrotrapezoid nuclei, may be responsible for the effect of adenosine on the hypercapnic ventilatory response. Sustained hypoxia has a biphasic effect on breathing, initially causing tachypnea but later giving way to bradypnea (Lawson and Long, 1983; Vizek et al., 1987). Rising adenosine levels have been implicated in hypoxia induced hypoventilation (Lopes et al., 1994; Yan et al., 1995), a potential contributor to SUDEP (Tao et al., 2010).

### Adenosine and PGES

Postictal generalized EEG suppression refers to the period of time immediately following a seizure in which the frequency and amplitude of EEG activity across the cortex is decreased (Lhatoo et al., 2010; Theeranaew et al., 2018). PGES has been associated with respiratory disturbances, decreased oxygen saturation, and increased postictal immobility (Seyal et al., 2013; Kuo et al., 2016; Rheims et al., 2019). Evidence from animal models indicates that adenosine plays a causal role in PGES (Figure 2 and Table 2). In amygdala kindled rats, systemic pretreatment with an adenosine analog prolongs PGES (Rosen and Berman, 1985; Whitcomb et al., 1990). Pretreatment with the adenosine receptor antagonist caffeine reduces the duration of PGES following amygdala kindled seizures (Whitcomb et al., 1990). Caffeine pretreatment does not alter electrocerebral suppression following electroconvulsive therapy; however, it is not clear whether this would be true in epilepsy patients with spontaneous seizures (Rosenquist et al., 1994). Further evidence is needed to determine whether PGES in epilepsy patients is tractable to adenosinergic manipulation.

### Adenosine and Spreading Depolarization

Spreading depolarization is a slow-moving wave which temporarily silences neuronal activity in the affected tissue (Leo, 1944; Pietrobon and Moskowitz, 2014). Spreading depolarization occurs in a number of diseases but is typically studied in the context of migraines (Lauritzen, 1994; Lauritzen et al., 2011). Seizures have the capacity to generate spreading depolarization waves similar to those seen in migraine patients (Kramer et al., 2017; Ssentongo et al., 2017). Adenosine levels are increased in the wake of spreading depolarization waves (Kaku et al., 1994; Lindquist and Shuttleworth, 2012, 2014; Seidel et al., 2016). The increase in extracellular adenosine brought on by spreading depolarization contributes to the suppression of neuronal activity which persists after the wave of depolarization has passed (Canals et al., 2008; Lindquist and Shuttleworth, 2012, 2017). Unlike in migraines, seizure-induced spreading depolarization can spread to the brainstem (Aiba and Noebels, 2015; Loonen et al., 2019). The propagation of spreading depolarization waves into the brainstem has been implicated as a causal factor in seizure-induced respiratory arrest and death (Aiba and Noebels, 2015; Aiba et al., 2016; Loonen et al., 2019). Whether increases in adenosine associated with seizure-induced spreading depolarization exacerbate postictal neuronal dysfunction or contribute to seizure-induced respiratory arrest has not been empirically investigated (Figure 2 and Table 2).

### Potential Proconvulsant Effects of A_2A_ Receptor Activation

Though increases in extracellular adenosine are generally anticonvulsant through A_1_ receptor activation, there are some findings that suggest that under certain circumstances A_2A_ receptor activation can be proconvulsant, but evidence for this is mixed. Hippocampal microinjection of an A_2A_ receptor agonist increased afterdischarge duration following piriform cortex kindled seizures in rats (Zeraati et al., 2006; Hosseinmardi et al., 2007); however, in this same model, microinjection of an A_2A_ receptor antagonist did not decrease afterdischarge duration (Zeraati et al., 2006; Hosseinmardi et al., 2007). The use of caffeine, a non-selective adenosine receptor blocker, reveals a more nuanced balance between the proconvulsant effect of A_2A_ receptor activation and the opposing effects of A1 receptor activation (Fredholm et al., 1999; El Yacoubi et al., 2008). Indeed, chronic caffeine administration decreased the susceptibility to chemoconvulsants in mice, an effect that involved A_2A_ receptor blockade (El Yacoubi et al., 2008). The neuroprotection from preventing A_2A_ receptor activation was further confirmed using transgenic mice lacking A_2A_ receptors, which were more resistant to pentylenetetrazol-induced seizures (El Yacoubi et al., 2008).

In a hyperthermia model of seizure induction, the threshold for seizure development in young rats was decreased by pretreatment with an A_2A_ receptor agonist and increased by A_2A_ receptor antagonist pretreatment (Fukuda et al., 2011). These findings indicate a proconvulsant effect of A_2A_ receptor activation; however, data collected in audiogenic seizure models indicated a primarily anticonvulsant effect of A_2A_ receptor activation (De Sarro et al., 1999; Huber et al., 2002). In contrast, other studies did not find any effects of A_2A_ receptor activation on seizure activity (Young and Dragunow, 1994; Rezvani et al., 2007; Uzbay et al., 2007; Hargus et al., 2012; Akula and Kulkarni, 2014). Additional evidence is necessary to clarify the conditions under which the A_2A_ receptor has proconvulsant effects and whether these effects are significant to SUDEP (Figure 2 and Table 2).

### Sleep Deficits

Epilepsy and sleep are interconnected, with one affecting the other (Kotagal and Yardi, 2008; Lanigar and Bandyopadhyay, 2017). Poor sleep is known to act as a trigger for certain forms of epilepsy such as nocturnal frontal lobe epilepsy, benign epilepsy with centrotemporal spikes, and Panayiotopoulos syndrome. On the other hand, having epilepsy can contribute to sleep disturbances and disorders such as insomnia and obstructive sleep apnea (Bazil, 2003; Staniszewska et al., 2017). In this section, we highlight the role of acute seizure-induced surges in adenosine in sleep/wake regulation (Bjorness and Greene, 2009). High adenosine levels promote sleep by inhibiting cholinergic neurons in the basal forebrain (Porkka-Heiskanen et al., 1997). Consistent with this notion, manipulation of ADK affected sleep regulation in mice (Palchykova et al., 2010). Using Kv1.1 knockout mice, a model of temporal lobe epilepsy with comorbid sleep disorders, Warren et al. (2018) demonstrated that surges in adenosine in the dorsal hippocampus and lateral hypothalamus contributed to lower seizure threshold and chronic partial sleep deprivation, respectively. Taken together, these studies suggest that adenosine dysregulation in chronic epilepsy may be responsible for the sleep disruption and sleep-related co-morbidities observed in epilepsy patients (Figure 2 and Table 2; Boison and Aronica, 2015). It is interesting to note that there is a strong association of SUDEP with sleep, with ∼ 70% of SUDEP-related deaths occurring during sleep (Ryvlin et al., 2013; Ali et al., 2017). Hence, investigating the relationship between sleep, adenosine, and epilepsy, particularly in the context of SUDEP, may yield significant insights into the pathophysiology of SUDEP.

## The Deadly

### The Adenosine Hypothesis of SUDEP

In 2010, it was observed in a kainic acid rodent seizure model that upregulating adenosine tone by inhibiting its metabolism had the seemingly paradoxical effect of initially preventing seizure activity, but then causing death when a seizure did occur (Shen et al., 2010). To explain this counterintuitive finding and, hopefully, to gain insights into the pathophysiology of SUDEP the adenosine hypothesis of SUDEP was formulated. The adenosine hypothesis of SUDEP suggests that seizure-induced increases in extracellular adenosine result in excessive inhibition of brain areas that are necessary for breathing which precipitates terminal respiratory arrest (Figure 1 and Table 3; Shen et al., 2010). This adenosine based explanation of SUDEP causally links the well-known increase in adenosine during and after seizures to respiratory failure.

**TABLE 3:** Experimental evidence which directly supports the adenosine hypothesis of SUDEP.

**The Deadly:** Seizure-induced increases in extracellular adenosine may precipitate SUDEP by excessive inhibition of brain areas that are necessary for breathing.		
**Reference**	**Seizure model**	**Core findings**
Shen et al., 2010	Kainic acid in unanesthetized mice	Increasing adenosinergic tone by inhibiting adenosine metabolism initially prevented seizure activity, but later precipitated seizure-induced death. This mortality was delayed by an adenosine receptor antagonist.
Ashraf et al., 2020	Kainic acid in anesthetized and tracheostomized rats	Seizure-induced death was only observed in rats with inhibited adenosine metabolism. Death was the result of central respiratory arrest as opposed to cardiac failure or laryngospasm. Impaired adenosine metabolism during seizures resulted in abnormal partial phrenic nerve bursts which were reduced by treatment with an adenosine receptor antagonist.
Faingold et al., 2016	DBA/2 audiogenic seizures	Pharmacological inhibition of adenosine metabolism increased the likelihood of seizure-induced death. Non-selective and A_2A_ specific adenosine receptor antagonism decreased the likelihood of seizure-induced death.
Kommajosyula et al., 2016	GEPR-9 audiogenic seizures	Inhibition of adenosine metabolism prolonged postictal motor impairment, exacerbated respiratory dysfunction, and increased the probability of death.

The adenosine hypothesis of SUDEP has significant explanatory power regarding the timing of respiratory arrest seen in SUDEP cases. As mentioned in the introduction, the best data currently available on the sequence of events which trigger SUDEP comes from a case series of SUDEP occurring in epilepsy monitoring units (Ryvlin et al., 2013). A consistent, but perplexing observation in these instances of SUDEP is that terminal respiratory failure began in the postictal period. In other words, the seizure ended, the patient was breathing for a period of 1–10 min, then the patient stopped breathing (Ryvlin et al., 2013). In this investigation, breathing was assessed by chest excursions observed by video along with the EEG artifacts associated with breathing (Ryvlin et al., 2013). This is not the most reliable method of respiratory measurement, particularly in situations where the view of the camera might be obstructed with bedding. Furthermore, quantification of tidal volume is not possible using video and EEG artifacts leaving open the possibility of severe hypoventilation prior to the terminal apnea. Nevertheless, the fact remains that the patients were breathing in the postictal period prior to the onset of fatal respiratory arrest (Ryvlin et al., 2013). This observation raises the following question: why do terminal apneas that are caused by seizures emerge when the seizure is over instead of during the seizure or at the end of the seizure? One possible clue that might be useful in answering this question is that the peak of seizure-induced adenosine surging occurs during the postictal period, not during the seizure itself (Van Gompel et al., 2014). If rising adenosine levels were responsible for the seizure-induced respiratory arrest seen in SUDEP one would expect terminal apnea to appear during the postictal period, when adenosine levels are at their highest. This prediction is borne out by clinical observations of SUDEP (Ryvlin et al., 2013).

### Evidence Concerning the Role of Adenosine in SUDEP

In a kainic acid mouse seizure model, the effects of impaired adenosine clearance were investigated by inhibition of the enzymes responsible for adenosine degradation, adenosine deaminase and ADK (Shen et al., 2010). Mice were pretreated with the adenosine deaminase inhibitor erythro-9-(2-hydroxy-3-nonyl)-adenine hydrochloride (EHNA) and the ADK inhibitor 5-iodotubercidin (5-ITU) prior to seizure induction *via* kainic acid. All mice with pharmacologically impaired adenosine clearance underwent seizure-induced death, whereas there was no mortality in the animals that received a saline injection prior to seizure induction. To ascertain whether the observed mortality was due to excessive adenosine signaling, the adenosine receptor antagonist caffeine was administered after the onset of seizure activity in animals treated with kainic acid and inhibitors of adenosine clearance. It was observed that caffeine treatment delayed death, supporting the hypothesis that excessive adenosine surging may play a causal role in the seizure-induced death phenotype (Shen et al., 2010). A limitation of this investigation is the lack of respiratory, cardiac, and electrocerebral quantification which preclude any conclusions regarding the cause of death.

In a more recent study, a similar approach of pharmacological suppression of metabolic adenosine clearance prior to kainic acid seizure induction was taken in rats (Ashraf et al., 2020); however, in this investigation concomitant measurements of EEG, heart rate, blood pressure, and phrenic nerve activity were made to clarify the cause of death. Furthermore, the rats in this study were tracheostomized prior to seizure-induction to rule out the possibility of laryngospasm. Seizure-induced laryngospasm is difficult to differentiate from seizure-induced central apnea and has been hypothesized to contribute to SUDEP pathophysiology (Nakase et al., 2016; Stewart et al., 2017; Budde et al., 2018; Irizarry et al., 2020). Seizure-induced death was observed in animals treated with 5-ITU and kainic acid, but not in animals treated with 5-ITU or kainic acid alone. Suppression of phrenic nerve activity preceded EEG flattening, cardiovascular failure, and death indicating a primarily respiratory cause of death. Unexpectedly, 5-ITU and kainic acid administration resulted in abnormal partial phrenic nerve bursts. These partial bursts were reduced by treatment with caffeine suggesting that they were related to excessive adenosine signaling. The precise cause of these partial phrenic nerve bursts and whether they occur in other seizure models has yet to be determined (Ashraf et al., 2020).

Audiogenic seizures in DBA/2 mice are a frequently used and well characterized model of seizure-induced death (Tupal and Faingold, 2006; Faingold et al., 2011; Irizarry et al., 2020). When subjected to a high intensity broadband acoustic stimulus, susceptible DBA/2 mice experience seizures that can evolve into seizure-induced respiratory arrest and death. Inhibition of adenosine metabolism by 5-ITU pretreatment was associated with an increased incidence of seizure-induced respiratory arrest. Conversely, caffeine pretreatment reduced the incidence of seizure-induced respiratory arrest. These findings indicate that excessive adenosine signaling may contribute to seizure-induced death in this model. Pretreatment with SCH 442416, an A_2A_ receptor antagonist, reduced the incidence of seizure-induced death. On the other hand, the A_1_ receptor antagonist DPCPX did not alter the probability of seizure-induced death suggesting that excessive A_2A_ receptor activation is the driving force in the effect of adenosine on vulnerability to seizure-induced death (Faingold et al., 2016).

Like DBA/2 mice, genetically epilepsy-prone rats (GEPR-9s) experience seizures and seizure-induced respiratory disruption following exposure to a high intensity broadband acoustic stimulus (Faingold, 1988). Seizures in GEPR-9s result in a period of postictal immobility, as indicated by a loss of the righting reflex, and respiratory disruption (Jobe et al., 1995; Kommajosyula et al., 2016). Seizures in GEPR-9s sometimes result in death; however, unlike the DBA/2 mouse, death is uncommon and does not immediately occur after the seizure (Kommajosyula et al., 2016). In this model, pharmacological inhibition of adenosine clearance by co-administration of EHNA and 5-ITU prolonged postictal motor impairment, exacerbated respiratory dysfunction, and increased the probability of death (Kommajosyula et al., 2016).

## Discussion

In summary, the influence of adenosine signaling in the context of epilepsy is nuanced and cannot be indiscriminately categorized as either beneficial or harmful (Figure 2 and Tables 1–3). Insufficient adenosine signaling results in inadequate neuronal inhibition, increased vulnerability to seizures, and the potentially fatal outcome of status epilepticus (Dragunow et al., 1985; Murray et al., 1985; Kochanek et al., 2006); however, paradoxically, excessive adenosine signaling may worsen periictal breathing, exacerbate PGES, and potentiate SUDEP (Rosen and Berman, 1985; Shen et al., 2010; Faingold et al., 2016; Kommajosyula et al., 2016). Though our understanding of the role of adenosine in epilepsy is rapidly improving there are still many unresolved questions and weaknesses in the existing literature that impede the development of adenosine-based therapeutic strategies to prevent SUDEP.

The adenosine hypothesis of SUDEP is largely predicated on the assumption that seizure-induced adenosine surging occurs in the brainstem, where adenosine is known to suppress breathing (Figure 1; Douglas et al., 1982; Yan et al., 1995; Gettys et al., 2013). Seizure-induced adenosine surging has primarily been studied in the context of seizure termination as opposed to seizure-induced death. As a result, seizure-induced adenosine surging has been identified in the hippocampus (Figure 1; During and Spencer, 1992; Berman et al., 2000; Aden et al., 2004; Etherington et al., 2009) and the cortex (Schrader et al., 1980; Van Gompel et al., 2014), but never directly measured in the brainstem. Characterizing the peri-ictal changes in adenosine levels in brainstem respiratory nuclei and nuclei previously implicated in SUDEP pathophysiology will be crucial to validating the adenosine hypothesis of SUDEP (Figure 1).

As discussed in the main body of this review, there are a number of mechanisms responsible for activity-dependent adenosine release. The relative contributions of these mechanisms appear to be regionally dependent. The mechanisms of activity dependent changes in adenosine signaling have primarily been studied using spontaneously occurring or electrically evoked neuronal activity. Insights gained regarding the mechanisms of activity dependent adenosine release in the context of evoked or naturally occurring neural activity may, or may not, be generalizable to seizures. Future investigations should examine the mechanisms underlying seizure-induced adenosine release and elucidate the spread of adenosine wave to the brainstem. Spreading depolarization waves result in an increase in extracellular adenosine which prolongs neuronal dysfunction (Canals et al., 2008; Lindquist and Shuttleworth, 2012, 2017); however, whether seizure-induced spreading depolarization waves elevate extracellular adenosine levels or whether such and increase might contribute to SUDEP is unknown.

Anatomically, where adenosine acts to potentiate SUDEP is unknown. Adenosine suppresses breathing in a variety of brainstem sites (Douglas et al., 1982; Yan et al., 1995; Gettys et al., 2013); however, direct evidence on seizure-induced alteration in adenosine levels in these brain areas is lacking. Deficits in serotonergic neurotransmission have been consistently implicated in SUDEP pathophysiology (Richerson and Buchanan, 2011; Faingold et al., 2014; Massey et al., 2014; Zhan et al., 2016; Petrucci et al., 2019). The mechanisms responsible for periictal suppression of serotonergic activity are unknown. The inhibition of serotonin neurons during and after seizures may be the result of adenosine surging; however, this has not been empirically investigated.

In addition to the inhibitory effect of adenosine on serotonergic neurotransmission, which is discussed in more detail in the “*Adenosine and breathing*” subsection, adenosine modulates the signaling of a number of other neurotransmitter systems which are relevant to epilepsy and seizure-induced death. Presynaptic A_1_ receptor activation inhibits the synaptic release of glutamate in brain areas notable for seizure activity, such as the hippocampus (Burke and Nadler, 1988). In contrast, A_2A_ receptor activation facilitates hippocampal and striatal glutamate release ostensibly by diminishing the inhibitory influence of A_1_ receptor activation (Popoli et al., 1995; Lopes et al., 2002). Correspondingly, A_1_ receptor antagonism increases glutamate release (Di Iorio et al., 1996; Quarta et al., 2004), whereas A_2A_ receptor antagonism decreases it (Popoli et al., 2003; Quarta et al., 2004).

Similar to glutamate, GABA release is decreased by A_1_ receptor activation and increased by A_2A_ receptor activation (Jeong et al., 2003; Hong et al., 2005; Yum et al., 2008). A_2A_ receptor mediated excitation of GABA releasing neurons in the respiratory brainstem has been used to explain the observation that both A_1_ and A_2A_ receptor agonists suppress breathing, despite their divergent effects on neuronal excitability (Wilson et al., 2004; Mayer et al., 2006).

Like serotonin, the monoaminergic transmitter norepinephrine may be protective against seizure-induced death. The norepinephrine reuptake inhibitor, atomoxetine, reduces the likelihood of seizure-induced death following maximal electroshock and audiogenic seizures (Zhang et al., 2017; Zhao et al., 2017; Kruse et al., 2019). Adenosine suppresses neuronal activity in the locus coeruleus (Shefner and Chiu, 1986) and focally inhibits norepinephrine release in the cortex (Harms et al., 1978; Taylor and Stone, 1980). Whether adenosinergic inhibition of norepinephrine neurons alters vulnerability to seizure-induced death has yet to be empirically investigated.

Arousal promoting cholinergic structures in the basal forebrain and brainstem are inhibited by adenosine (Rainnie et al., 1994; Porkka-Heiskanen et al., 1997; Peng et al., 2020). Seizures suppress the activity of cholinergic neurons in the basal forebrain and pedunculopontine tegmental nucleus (Motelow et al., 2015). Given the hypothesized role of the ascending arousal system in the prevention of SUDEP (Massey et al., 2014), the role of adenosinergic inhibition of the cholinergic system during seizures should be investigated.

Most of the experimentation pertinent to the role of adenosine in seizure-induced death has been conducted in acute seizure models, often in seizure-naïve animals. Epileptogenesis and the occurrence of repeated seizures alters the brain in ways that might be meaningful to SUDEP; for instance, an altered expression/function of ADK and adenosine receptors has been noted during epileptogenesis (Patodia et al., 2020). Therefore, it would be beneficial to use models of epilepsy which feature spontaneous seizures and spontaneous seizure-induced death for investigating SUDEP. To this end, *Kcna1^–/–^* mice, which lack voltage-gated Kv1.1 channels, experience spontaneous seizures and undergo seizure-induced death at approximately postnatal day 50 (Moore et al., 2014). Dravet syndrome is a severe infantile onset epilepsy with a high rate of SUDEP (Dravet, 1978; Genton et al., 2011). Dravet syndrome is the result of mutations in the *Scn1a* gene which encodes the voltage-gated sodium channel Nav1.1. Mice with similar mutations display phenotypes comparable to those seen in Dravet syndrome, including seizure-induced death which might prove to be an effective tool for SUDEP investigations (Ito et al., 2013; Kalume et al., 2013; Kim et al., 2018).

A growing body of evidence implicates adenosine signaling in a variety of adverse seizure outcomes such as respiratory suppression, PGES, and SUDEP (Figure 2 and Tables 2, 3); however, it is not yet clear how this information can be leveraged to inform clinical preventative strategies. Systemic adenosine receptor antagonism may reduce vulnerability to SUDEP, but there are a number of reasons why this might not be a viable clinical option. Most cases of SUDEP occur during the night, presumably while the patient is asleep (Lamberts et al., 2012; Ali et al., 2017; Purnell et al., 2018). An adenosine antagonist, such as caffeine, taken before bed would be likely to disrupt the patients sleep (Drake et al., 2013). Sleep disruption can result in a variety of adverse health outcomes including an increased likelihood of seizures (Bennett, 1963; Mendez and Radtke, 2001; Medic et al., 2017). Because it is generally agreed that SUDEP occurs consequent to a seizure (Ryvlin et al., 2013; Massey et al., 2014), anything that might impair a patients seizure control should be avoided. Furthermore, adenosine receptor antagonists can prolong seizures potentially increasing the amplitude of the seizure-induced adenosine surge (Dragunow and Goddard, 1984; Bozymski et al., 2018). The beneficial effect of antagonizing adenosine receptors might be counteracted by a higher surge in extracellular adenosine following seizure termination. Lastly, chronic administration of adenosine receptor antagonists can result in increased adenosine receptor expression (Fredholm, 1982). An increase in adenosine receptor expression, particularly in the brainstem, might increase vulnerability to seizure-induced respiratory arrest.

Sudden unexpected death in epilepsy typically occurs during sleep (Ali et al., 2017). Given the role of changing adenosine concentrations in sleep/wake regulation (Basheer et al., 2004) and the adenosine hypothesis of SUDEP outlined in this review, the reader may arrive at the conclusion that differential adenosine concentrations during sleep might be in some way related to the increased incidence of SUDEP during sleep. It should be noted that the increase in adenosine over the course of wakefulness is primarily localized to the basal forebrain and is absent in the dorsal raphe, an arousal promoting brainstem nucleus which has been implicated in SUDEP (Porkka-Heiskanen et al., 2000; Zhang et al., 2018; Petrucci et al., 2020). Furthermore, extracellular adenosine concentrations quickly fall during sleep to levels lower than those seen during wakefulness (Porkka-Heiskanen et al., 1997). Thus, it is not clear whether fluctuations in adenosine are related to the increased rate of SUDEP during sleep.

By improving our understanding of periictal adenosine dynamics and developing novel strategies for influencing adenosinergic signaling we may gain insights into how seizures and their most tragic sequelae can be prevented. Therapeutically, adenosine augmentation strategies are some of the most effective strategies for seizure control (Gouder et al., 2003; Boison, 2012), however, SUDEP risk needs to be considered and local adenosine augmentation strategies might be the most effective (Figure 1; Boison, 2009). In addition to the benefits noted here, adenosine augmentation might also help improve affective, psychiatric, and cognitive impairments (Boison and Aronica, 2015); co-morbidities that are prevalent among patients with epilepsy (Gaitatzis et al., 2004; LaFrance et al., 2008). Most importantly, novel findings show that adenosine therapy can prevent epilepsy development through an epigenetic mechanism (Williams-Karnesky et al., 2013; Lusardi et al., 2015; Sandau et al., 2019). Those strategies employed only transiently in a pre-epileptic brain are not expected to be associated with increased SUDEP risk, rather it is intended to avert epilepsy, and thereby prevent the primary antecedent to seizure-induced death.

## Author Contributions

All authors listed have made a substantial, direct and intellectual contribution to the work, and approved it for publication.

## Conflict of Interest

The authors declare that the research was conducted in the absence of any commercial or financial relationships that could be construed as a potential conflict of interest.

## Abbreviations

5-ITU: 5-iodotubercidin
A_1/2A_: adenosine receptor
ADK: adenosine kinase
ATP: adenosine triphosphate
DPCPX: A_1_ receptor antagonist
dnSNARE: N-ethylmaleimide -sensitive factor attachment protein receptor
EEG: electroencephalogram
EKG: electrocardiogram
EHNA: erythro-9-(2-hydroxy-3-nonyl)-adenine hydrochloride
GEPR-9s: genetically epilepsy-prone rats
GRAB_*Ado*_: GPCR-activation based adenosine sensor
PGES: postictal generalized EEG suppression
SCH 442416: A_2 A_ receptor antagonist
SUDEP: sudden unexpected death in epilepsy.
